# Remibrutinib inhibits hives effector cells stimulated by serum from chronic urticaria patients independently of FcεR1 expression level and omalizumab clinical response

**DOI:** 10.1002/clt2.12227

**Published:** 2023-03-04

**Authors:** Ramón Gimeno, Clara Ribas‐Llauradó, David Pesque, Evelyn Andrades, Bruno Cenni, Barbara Ambros, Ramon Pujol, Ana M. Giménez‐Arnau

**Affiliations:** ^1^ Laboratory of Immunology Department of Pathology Hospital del Mar Barcelona Spain; ^2^ Department Immnology Hospital del Mar Medical Research Institute (IMIM) Barcelona Spain; ^3^ Department of Medicine and Life Sciences Universitat Pompeu Fabra Barcelona Spain; ^4^ Department of Dermatology Hospital del Mar IMIM Universitat Pompeu Fabra Barcelona Spain; ^5^ Department of Research BC Novartis Institutes for BioMedical Research, Novartis Basel Switzerland; ^6^ Department of Clinical Development BA Global Drug Development, Novartis Basel Switzerland

**Keywords:** basophils, Bruton's tyrosine kinase, chronic urticaria, IgE, mast cells, omalizumab, remibrutinib

## Abstract

**Background:**

Despite advances in the treatment of chronic urticaria, in a significant percentage of the patients symptoms are not fully controlled with conventional approaches. New strategies under development include blocking intracellular mediators of mast cell and basophil activation.

**Objective:**

We aim to investigate the effects of the Bruton's tyrosine kinase (BTK) inhibitor remibrutinib on human blood basophils and CD34^+^‐derived mast cells activation induced by serum obtained from chronic urticaria patients.

**Methods:**

Twenty‐two patients with chronic spontaneous urticaria (mean age 52 years, 27% women) and 22 patients with chronic inducible urticaria (46 years, 27% women) were included in the study together with a sex‐matched control group. Patients were classified as responders or non‐responders to anti‐IgE therapy on the basis of their clinical data, FcεR1a expression on blood basophils and total IgE levels. Changes on CD63 expression—as an activation marker‐, were used to evaluate in vitro the response of basophils and mast cells to serum exposure and the inhibitory effects of remibrutinib.

**Results:**

Remibrutinib inhibits degranulation induced by IgE cross‐linking in mast cells and basophils and also the activation triggered by factors present in the sera of spontaneous and inducible chronic urticaria patients. Patient's serum induces a greater degranulation of effector cells than controls. Activation of mast cells and basophils by patient sera and remibrutinib effects were not related to omalizumab responsiveness.

**Conclusion:**

Remibrutinib inhibits activation of human basophils and mast cells induced in vitro by exposure to the serum of chronic urticaria patients independently of their response to omalizumab.

## 1 INTRODUCTION

Urticaria is a common cutaneous and systemic condition characterized by the presence of intensely pruritic, well circumscribed, raised wheals of variable size and shape. In both chronic and acute urticaria, angioedema can be concomitantly present with hives in about a 40% of cases. Although often self‐limited and benign, hives can be very uncomfortable, interfering with sleep and normal daily activity. This is especially relevant considering that in up to 30%–40% of the patients' symptoms may persist for years. In chronic urticaria (CU), lesions appear and recur over the course of 6 weeks or more. In chronic spontaneous urticaria (CSU), which accounts for the vast majority (80%) of the cases of CU, specific external triggers are absent. In contrast, in chronic inducible urticaria (CIndU), physical or environmental stimuli such as cold, heat, exercise, pressure, sunlight, or vibration are responsible for the appearance of itchy wheals and/or angioedema.^1^

The different subtypes of CU (and angioedema) have as a common pathophysiologic mechanism the release by mast cells and basophils of histamine and other proinflammatory mediators. Degranulation may be triggered by immunoglobulin E (IgE) and/or immunoglobulin G (IgG) autoantibodies against IgE or the high‐affinity IgE receptor. High‐affinity receptor (FcεR1) binds the Fc region of IgE. FcεRI crosslink with IgE‐autoallergen, IgE–IgG, IgG, or IgM. These mechanisms were suggested in CU, both CSU and CIndU. It is well‐known also that non‐IgE and nonimmunologic mast cell activation can occur through different mechanisms.^1^ In fact, many other receptors including, MRGPRX2, C5aR, PAR‐1, PAR‐2, CRTh2, and cytokine receptors can be activated by various signals when hives are present.^2^

Antihistamines are the mainstay treatment for CU. In non‐responsive patients, biologic therapy with anti‐IgE antibodies (omalizumab) is indicated.^3^ Still in a significant percentage of patients it is not possible to achieve a complete control of the symptoms, highlighting the importance of exploring new therapeutic targets.^4^

Bruton's tyrosine kinase (BTK) is a cytoplasmic kinase expressed in B‐cells and macrophages but also in mast cells/basophils.^5^ BTK is essential for signaling through FcεR1. Remibrutinib, a covalent, oral BTK inhibitor,^6^ inhibited basophil activation in healthy volunteers,^7^ and has shown efficacy in patients with moderate to severe CSU in a phase 2b trial.^8^

There is also a growing need for biomarkers that can help to assess the severity and activity of CU and predict response to treatment. It has been previously demonstrated that the basophil baseline total IgE and FcεR1a expression can be used to characterize the response to omalizumab in CSU and CIndU patients.^9^, ^10^, ^11^, ^12^ Mast cells and basophils are key effector cells in the pathogenesis of CU. Using as relevant targets human hematopoietic stem cell‐derived mast cells and basophils from peripheral blood, we aim to investigate the effects of the BTK inhibitor remibrutinib on in vitro cell activation induced by serum obtained from CSU and CIndU patients.

## 2 METHODS

### 2.1 Patients

Adult patients suffering from CU at the Urticaria Clinic of the Department of Dermatology of Hospital del Mar (Barcelona, Spain) were included in the study. Specifically, we included patients with CSU with disease duration of more than 3 months and with an urticaria activity score (UAS7) defined at least as moderate (UAS7 ≥ 16; itch ≥ 8). In the case of pure CIndU, a positive result for each standardized provocation test was required. For CSU, the UAS7 and the urticaria control test (UCT) were used to validate patient reported outcomes and to assess efficacy of the treatment. For CIndU, besides the UCT, specific thresholds, as defined in the guidelines,^13^ were assessed at baseline and after 6 months of treatment. Treatment included second generation H1‐antihistamines combined with anti‐IgE (omalizumab) therapy to establish the control of the disease. In total, 44 patients were included (22 with CSU and 22 with CIndU).

Peripheral blood samples from patients with CU were analyzed before the initiation of omalizumab therapy to assess both total IgE serum levels and the FcεR1a expression on blood basophils. According to their clinical response to omalizumab, IgE and FcεR1a receptor expression on blood basophils, patients were classified as responders or non‐responders to anti‐IgE therapy using thresholds previously established.^9^, ^11^ CSU patients who were clinically non‐responders to omalizumab despite increasing the dose of the drug up to 600 mg, did not obtain a UAS7 <16, UCT > 12, and did not reduce their baseline UAS7 after 6 months of treatment. The dose of omalizumab was increased after the third administration due to not achieving a goal of reducing UAS7 to <16, UCT > 12, or a reduction in UAS7 after the third administration. CIndU patients non‐responders to omalizumab did not obtained UCT > 12 despite increasing the dose of the drug up to 600 mg. The dose of omalizumab was increased after the third administration if the goal of UCT>12 was not obtained.

In addition, peripheral blood samples from a control group of 22 sex‐equivalent healthy adult controls (HCs) with no family or personal history of allergic asthma, allergic rhinitis, CU, or atopic dermatitis were included as reference.

Exclusion criteria for study participation were: <18 years old, concomitant treatment with immunosuppressive agents and/or corticosteroids, and chronic pruritic diseases.

The present study has been carried out in accordance with The Code of Ethics of the World Medical Association (Declaration of Helsinki) for experiments involving humans. The local Clinical Research Ethics Committee granted ethical approval for the study (approval no. 2018/7915/I). Signed informed consent was obtained from all participants.

### 2.2 Mast cell generation

Mononuclear cells were obtained from umbilical cord blood (Banc de Sang i Teixits, Barcelona, Spain) by density gradient centrifugation and CD34^+^ progenitor cells purified by immunomagnetic selection using the CD34 MicroBead Kit (Miltenyi Biotec, Bergisch Gladbach, Germany). For mast cell generation, CD34^+^ cells were cultured at a concentration of 5 × 10^5^ cells/ml in StemSpan™ serum‐free expansion medium (SFEM) medium (StemCell Technologies, Vancouver, Canada) supplemented with 50 μg/ml of human low‐density lipoprotein (LDL) (Stemcell Technologies), interleukin 3 (IL‐3) and stem cell factor (SCF) (100 ng/ml each) and IL‐6 (250 ng/ml). At day 3, cell concentration was adjusted to maintain culture at 5 × 10^5^ cells/ml by adding fresh StemSpan SFEM supplemented with 50 μg/ml human LDL, IL‐3, and SCF (20 ng/ml each) and 100 ng of IL‐6. At day 7, cells were washed and resuspended in StemPro™‐34 serum‐free medium (SFM) (Thermofisher Scientific, Waltham, MA, United States [USA]) supplemented with SCF and IL‐6 (100 ng/ml each). These conditions were maintained during the next 5 weeks of culture, adjusting cell concentration by adding fresh medium supplemented with cytokines (all from Miltenyi Biotech). During the sixth week of culture and further, medium was enriched by adding a supplement of 10% fetal bovine serum (FBS). To monitor cell development, flow cytometry and cell numbers were determined weekly.

### 2.3 Mast cell stimulation

First, as a sensitization step, cells were incubated 48–72 h at 37°C with 1 μg/ml of human IgE (NBS‐C BioScience, Vienna, Austria) and 20 ng/ml IL‐4 (Peprotech, London, United Kingdom [UK]). After sensitization, cells were harvested, washed, counted, and resuspended in the stimulation buffer included in the Flow CAST^®^ basophil activation test (BAT) (Bühlmann, Schönenbuch, Switzerland).

To assess the potential of human serum to stimulate mast cells, sensitized cells (8–10 × 10^4^ cells/well) were exposed to controls' and patients' sera (10%) and incubated at 37°C. After 30 min, mast cell degranulation was stopped by placing the cells on ice for 10 min. To study the inhibitory effect of remibrutinib (LOU064, Novartis, Basel, Switzerland) on mast cells degranulation, cells were treated with 1 μM remibrutinib for 1 h at 37°C before adding the stimulus. In each experiment negative (sensitized cells without any stimulus) and positive controls (sensitized mast cells with 1 μg/ml goat anti‐human IgE [Abcam, Cambridge, UK]) were included.

### 2.4 Basophil stimulation

To study basophil activation, Flow CAST^®^ BAT was used following manufacturer's instructions. In these experiments, blood from healthy donors was incubated with or without 1 μM remibrutinib for 1 h at 37°C before the addition of controls' and patients' sera (10%). In each experiment, a negative control consisting of unstimulated blood and a positive control (blood stimulated with anti‐FcɛR1 monoclonal antibodies [mAb] to crosslink the receptor) were included. After an additional 30 min of incubation with the sera at 37°C, blood cells were stained with anti‐CD193 (chemokine receptor 3, CCR3)‐PE and the anti‐CD63‐fluorescein (FITC) included in the staining reagent of the kit. Finally, red blood cells were lysed, and samples were analyzed by flow cytometry.

### 2.5 Flow cytometry

Development of mast cells was monitored by flow cytometry using a standard protocol. Briefly, cells were harvested from the cultures at defined time‐points, incubated for 30 min with a saturating amount of human Igs to reduce non‐specific bindings and stained with anti‐CD117 (c‐kit)‐APC and anti‐FcɛR1a‐PerCP/Cy5.5 mouse mAbs for 30 min at 4°C in the dark. After that, cells were washed, fixed, and analyzed using a Becton Dickinson LSRII flow cytometer and the DIVA software (BD Biosciences, San Jose, CA, USA). Upon stimulation, mast cell activation was assessed, following a similar procedure, using an anti‐CD63‐BV421 antibody together with the above‐mentioned lineage‐specific antibodies (all the antibodies from BioLegend, San Diego, CA, USA).

### 2.6 Statistics

Descriptive analyses were performed for each clinical value using median and range for quantitative variables and absolute (*n*) and relative (%) frequencies for categorical variables. Mann–Whitney *U* test was used to compare the percentage of activation, degranulation, and inhibition in both basophils and mast cells in all comparisons performed.

All analyses were carried out with GraphPad Prism 8.0 software (GraphPad, La Jolla, California, USA) and a *p*‐value <0.05 was considered statistically significant.

## 3 RESULTS

### 3.1 Patient characteristics

**FIGURE 1:**
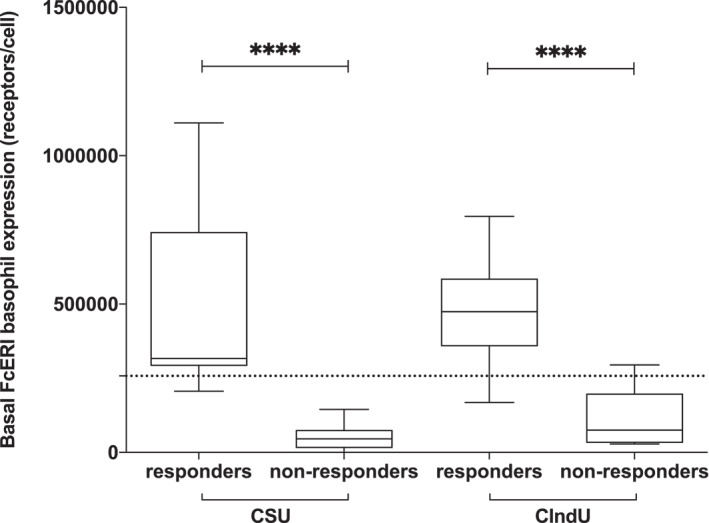
Classification of patients into omalizumab responders or non‐responders. It was done taking into consideration the baseline FcεR1 levels (expressed as number of receptors per cell) and a previously described threshold (see Section 2). Cases were divided into spontaneous (CSU) or inducible (CIndU) forms of chronic urticaria (CU). Box‐whiskers plots presenting median, interquartile range, maximum, and minimum of basophil high‐affinity IgE receptor (FcεR1) levels in all patients with CU included in the study. Mann–Whitney *U* test ****p < 0.0001.

Serum samples from 44 patients diagnosed with CU were collected (22 CSU patients and 22 pure CIndU patients). Patients suffering from CIndU included twelve cold urticaria (54.5%), three solar urticaria (13.6%), three symptomatic dermographism (13.6%), three cholinergic urticaria (13.6%), and one delayed pressure urticaria (4.5%). Demographic features of the study population are described in Table 1. Patients were classified as responder or non‐responder to anti‐IgE therapy based on the activity improvement (CSU), the control assessment (CSU, CIndU), and the FcεR1 baseline expression in basophils assessed by flow cytometry. CSU patients who respond to omalizumab showed a median FcεR1 expression density of 316,610 compared to those who do not respond to omalizumab, 45,700 (*p* < 0.0001). CIndU patients who respond to omalizumab showed a median FcεR1 expression density of 474,639 compared to those who do not respond to omalizumab, 75,627 (*p* < 0.0001) (see Figure 1).

**TABLE 1:** Clinical and demographic characteristics of the study population.

	CSU	CIndU
Cases, *n*	22	22
Female sex, *n* (%)	17 (77.27%)	17 (77.27%)
Age (range), years	52 (27–81)	46 (17–79)
Concomitant atopic features, *n* (%)	2 (9.10%)	3 (13.64%)
Angioedema, *n* (%)	13 (59.10%)	1 (4.45%)
Thyroid impairment, *n* (%)	9 (40.91%)	2 (9.10%)
Disease duration, median (range), months	46 (6–216)	128 (22–336)
Concomitant CIndU, *n* (%)	4 (18.18%)	2 (9.10%)
Serum IgE levels, median (range), kU/L	112.66 (1.0–966)	161.8 (2.6–821)
Basophil count, median (range), 10^3^/ml	0.02 (0.00–0.4)	0.03 (0.01–1.6)
FcεR1 expression, median (range), density	252,111 (0–111,0533)	312,779 (9264–671,816)
Omaluzimab response, *n*	9	14
Omaluzimab no response, *n*	13	8

Abbreviations: CIndU, chronic inducible urticaria; CSU, chronic spontaneous urticaria; IgE, immunoglobulin E.

### 3.2 Remibrutinib inhibits mast cell and basophil degranulation induced by IgE cross‐linking

**FIGURE 2:**
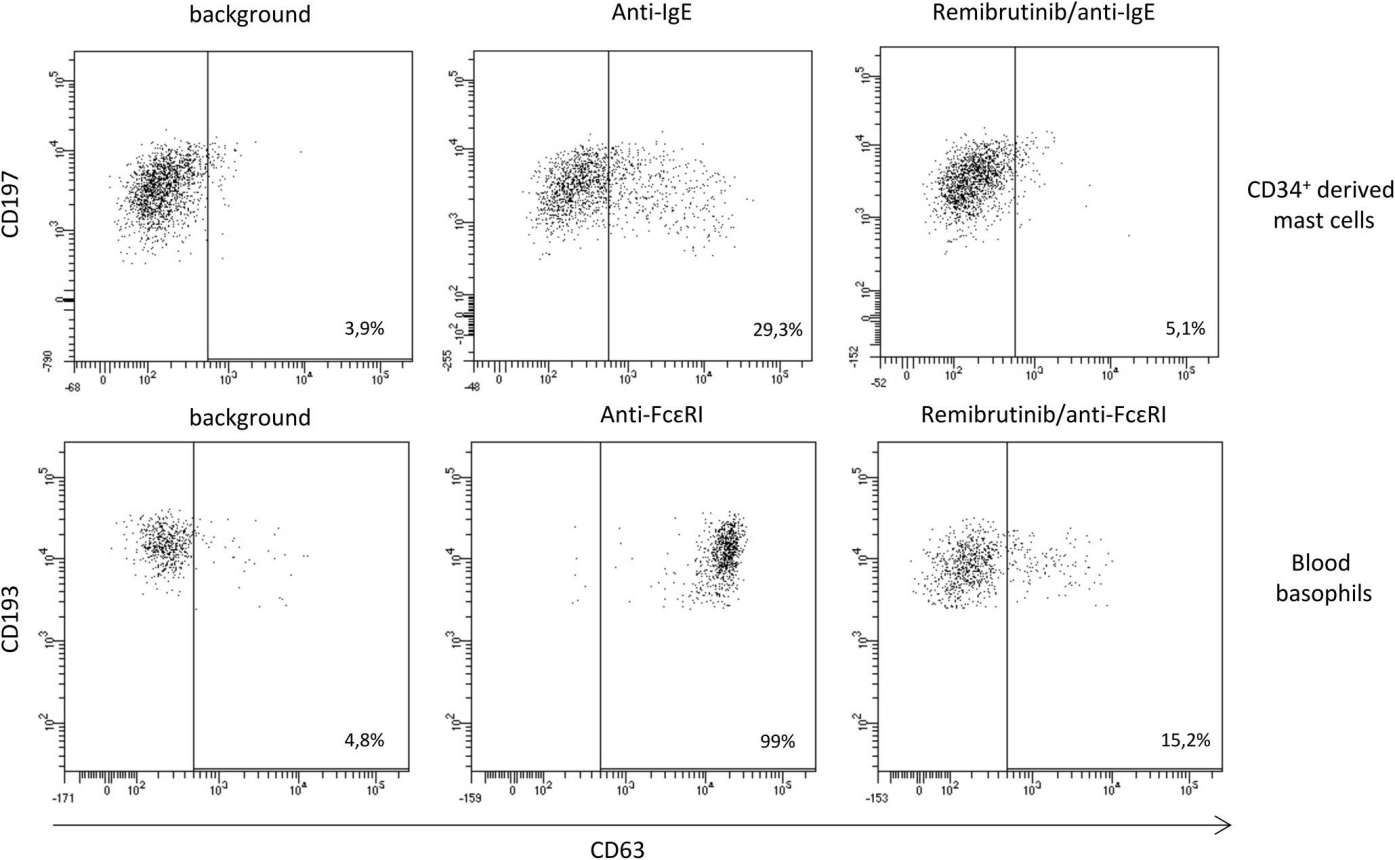
Remibrutinib inhibits effector cell activation induced by IgE crosslinking. CD34^+^‐derived mast cells (upper panels) or blood basophils (lower panels) were activated by IgE‐mediated (mast cells) or directly (basophils) through FcεR1 crosslinking and CD63 upregulation established by flow cytometry. Percentage of activated cells is indicated in the lower right corner of each plot. A preincubation step with remibrutinib reduces the frequency of activated cells. This is a representative experiment of more than five performed.

IgE cross‐linking is a very efficient trigger of mast cell and basophil activation. As mast cells are not found in circulation, a feasible alternative is to generate them from CD34^+^ human hematopoietic stem cells differentiated in vitro (see Supplementary Figure 1). The differentiation process can be followed by assessing the expression of CD117 (c‐kit) and the high‐affinity IgE receptor (FcεR1a) on the surface of the developing cells. To evaluate the ability of CD34^+^‐derived mast cells to respond to relevant stimuli, cells were incubated with anti‐IgE antibodies to induce the crosslinking of the FcεR1a and the initiation of a signaling cascade. As shown in Figure 2, CD63 expression, as a read‐out of activation and degranulation, is increased in the presence of IgE + anti‐IgE complexes. A previous treatment with remibrutinib results in a drastic reduction in the percentage of CD63^+^ mast cells and an almost complete abrogation of basophil activation induced by anti‐FcεR1 antibodies.

### 3.3 Serum from CU patients has a greater ability to induce mast cell and basophil degranulation than sera obtained from HCs

**FIGURE 3:**
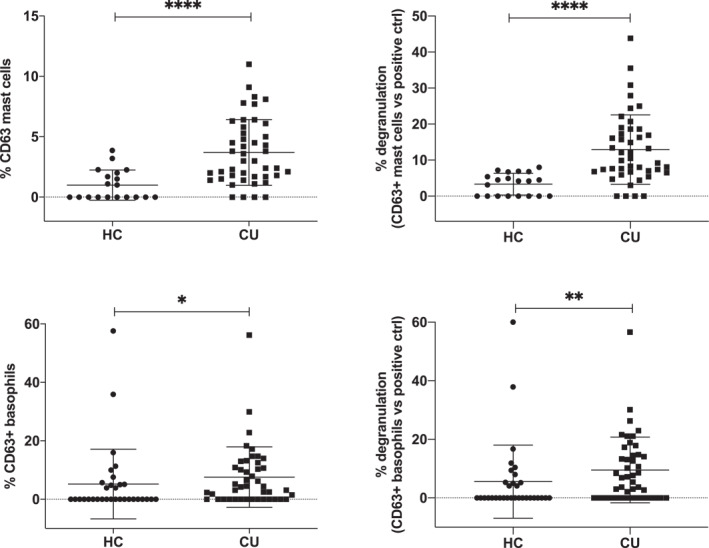
Activation of mast cells and basophils induced by CU patient serum. CD34^+^‐derived mast cells (upper panels) or blood basophils (lower panels) were incubated with serum obtained from healthy donors (HC) or from urticaria (CU) patients and the upregulation of CD63 established in the relevant population by using specific monoclonal antibodies and flow cytometry. Percentages of CD63‐expressing cells are shown in absolute terms (left panels) or percentage referred to the maximum activation obtained with the positive control (right panels). Mann–Whitney *U* test: *p < 0.05, **p < 0.01, ****p < 0.0001. Each dot in this figure represents the serum of a single individual.

The use of anti‐IgE therapies is a major breakthrough in the management of CU.^14^ It is now considered an elective choice for the treatment of antihistamine resistant CSU patients and it may be useful also in the management of some forms of CIndUs.^15^ In the former case, basal IgE levels or expression of FcεR1a could anticipate the response to IgE therapies. Although there are different pathogenetic aspects of the CIndUs that remain to be defined the autoimmune mechanism has been shown, for example, for cold urticaria or cholinergic urticaria. As a next step, we examined whether serum obtained from CU patients may have the ability to activate mast cells and/or basophils. In fact, as shown in Figure 3, sera from patients with CU were capable of inducing degranulation of mast cells (upper panels) and basophils (lower panels) to a greater extent than the factors contained in the sera of healthy individuals used as controls.

**FIGURE 4:**
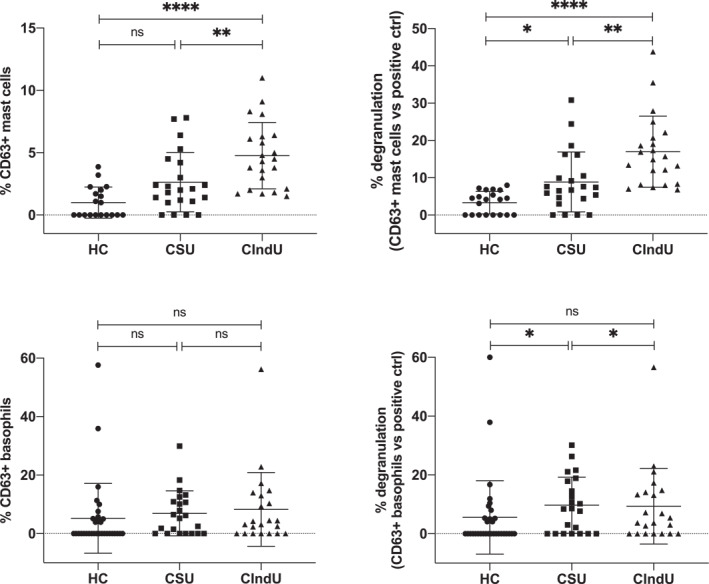
Mast cells are more sensitive than basophils to activation induced by factors contained in serum from CIndU patients. CD34^+^‐derived mast cells (upper panels) or blood basophils (lower panels) were incubated with serum obtained from healthy donors (HC) or from spontaneous (CSU) or induced (CIndU) chronic urticaria patients and the upregulation of CD63 established in the relevant population by using specific monoclonal antibodies and flow cytometry. Percentages of CD63‐expressing cells are shown in absolute terms (left panels) or percentage referred to the maximum activation obtained with a positive control (right panels). Mann–Whitney *U* test: ns no significant, *p < 0.05, **p < 0.01, ****p < 0.0001. Each dot in this figure represents the serum of a single individual.

When the two groups of CU patients (CSU and CIndU) were analyzed separately, at the population level and in these experimental settings sera from CIndU patients had greater power at stimulating mast cells than sera from CSU patients, whereas minimal differences were detected when basophils were used as cellular substrate (Figure 4 and Supplementary Figure 2). This interesting finding is not easily explained given the current lack of knowledge about the exact pathogeny of the different CIndUs. The findings suggest that potentially implicated circulating factors have a greater capacity to activate mast cells than those circulating in CSU. In fact, clinically the expression of CIndUs tends to be localized only in the skin where the triggering external stimulus acts and not generalized, involving mostly tissue mast cell.

### 3.4 Activation of Basophils and Mast cells by patient sera is not linked to Omalizumab responsiveness

**FIGURE 5:**
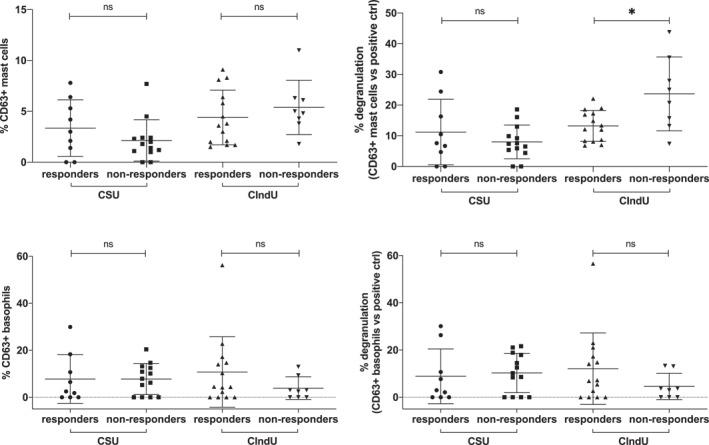
Mast cells and basophils activation induced by serum factors is not related to omalizumab responsiveness. CD34^+^‐derived mast cells (upper panels) or blood basophils (lower panels) were incubated with serum obtained from spontaneous (CSU) or induced (CIndU) chronic urticaria patients and the upregulation of CD63 established in the relevant population by using specific monoclonal antibodies and flow cytometry. Percentages of CD63‐expressing cells are shown in absolute terms (left panels) or percentage referred to the maximum activation obtained with a positive control (right panels). In these panels, patients are classified in responders or non‐responders to omalizumab therapy. Mann–Whitney *U* test: ns no significant, *p < 0.05. Each dot in this figure represents the serum of a single individual.

Patients who do not respond to antihistamines are presently treated with anti‐IgE therapies. Although this has become a big advance in the management of these patients, still a significant number of them do not respond to IgE blockers. It has been previously shown that measurement of FcεR1 expression levels on blood basophils can help to identify those patients that will not respond to omalizumab. Next, we studied whether the sera obtained from CSU and CIndU patients which respond to omalizumab (patients with and overexpression of FcεR1a) differ from sera obtained from non‐responders (patients with lower FcεR1a expression) in their ability to activate effector cells. Except for a major capability of sera from non‐responder CIndU patients to activate mast cells, there were not statistically significant differences among these groups (Figure 5). These findings support that other activating factors may be present in the serum and work independently of FcεR1, a relevant pathogenic aspect that has been suggested in previous studies. Thus in two mast cells lines expressing (LAD2) or lacking (HCM‐1) the IgE receptor, a pool of sera from patients with CSU able to induce mast cells degranulation in both types of cultures and curiously most of these patients showed autologous serum skin test negative.^16^ Likewise the ability to activate mast cells by low molecular weight serum fractions (100–50–30 kDa) from patients with CSU has been described.^17^

### 3.5 Remibrutinib inhibits in vitro basophil and mast cell degranulation induced by patient serum

**FIGURE 6:**
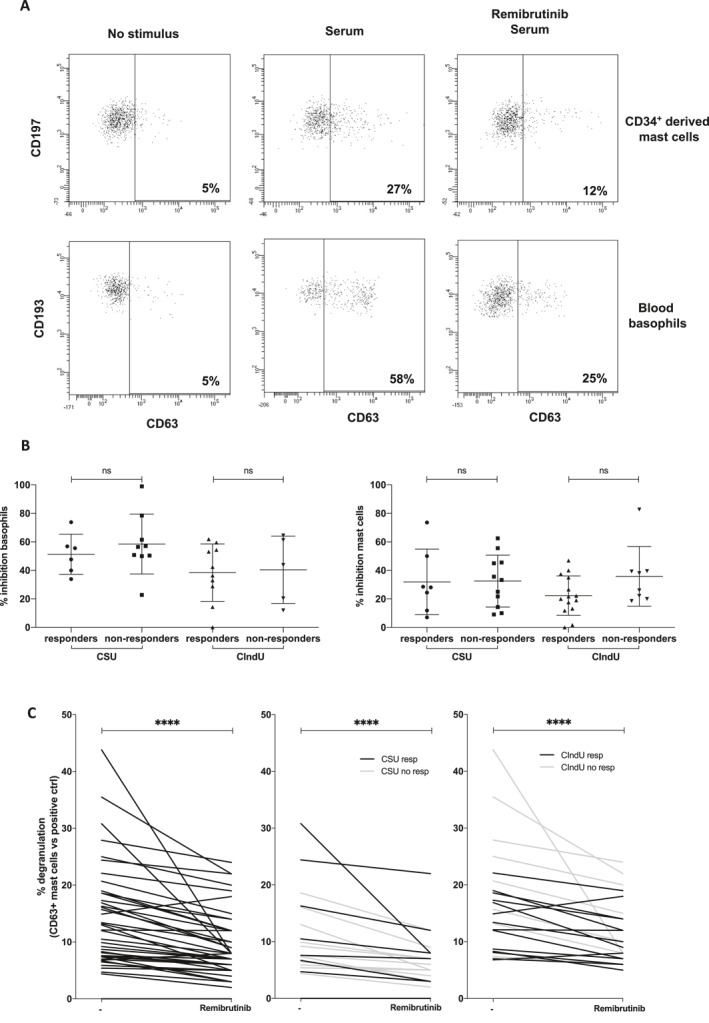
Remibrutinib inhibits effector cell activation induced by patient's serum exposure. (A), CD197^+^ and CD34^+^‐derived mast cells (upper panels) and CD193^+^ blood basophils (lower panels) were exposed to serum from CU patients and CD63 upregulation established by flow cytometry. Percentage of activated cells is indicated in the lower right corner of each plot. As shown in the right part of the panel, a preincubation step with remibrutinib reduces the frequency of activated cells. This is a representative experiment of more than five performed. (B) CSU or CIndU patients are classified in responders or non‐responders to omalizumab therapy and the inhibitory effect of remibrutinib in each situation assessed as indicated before. Mann–Whitney *U* test: ns no significant. Each dot in this figure represents the serum of a single individual. (C) Representation of the effect of BTK inhibition by remibrutinib on the activation induced by patient's serum using mast cells as cellular substrate. On the left all the CU patients are represented, in the middle panel only CSU patients are shown and on the right panels only data from CIndU patients are depicted. Patients are further characterized as responsive (resp) or not (no resp) to omalizumab. Percentage of inhibition is referred to the maximum obtained with a positive control (Wilcoxon test: ****p < 0.0001).

The positive effect of anti‐IgE therapies in the control of CU suggests a key involvement of IgE or the signaling triggered by its binding to the IgE receptors in the pathogenesis of the different types of urticaria. Remibrutinib inhibits basophil and mast cell activation induced by IgE cross‐linking; we wondered whether it is also capable to block activation promoted by serum exposure. As it is seen in Figure 6A,B, remibrutinib reduced expression of CD63 induced by serum of patients with CU on both basophils and mast cells.

Notably, at a population level, the blocking effect on CD63 expression seems to be significantly higher in basophils but similar between CSU and CIndU and independently if patients were responsive to omalizumab or not. When comparing the degree of inhibition of mast cell activation elicited by treatment case by case (Figure 6C), we found an effect both at the population and individual levels.

## 4 DISCUSSION

Despite of being a relatively frequent skin disorder, the pathogenesis of CSU is still unclear. Activation of mast cells and basophils via either the FcԑR1 receptor^18^ or by non‐immunological mechanisms (through other receptors such as MRGPRX2, neurotransmitters or physicochemical stimuli)^19^ has been described, with the release of inflammatory mediators and cytokines as the ultimate consequence of such activation. The presence of specific IgE antibodies against exogenous and endogenous antigens such as thyroid peroxidase, thyroglobulin, double‐stranded deoxyribonucleic acid (DNA), staphylococcal exotoxins or interleukin‐24 (Type I autoimmunity) or IgG autoantibodies against IgE or FcԑR1 (Type IIb autoimmunity) have been described in CSU patients^19^, ^20^, ^21^ and may induce degranulation of effector cells after crosslinking the FcԑRI. In contrast, in CIndU patients the presence of autoimmunity has not been proven yet but autoallergy was suggested in certain types. A transmissible serum factor was identified in cold urticaria^22^ and in cholinergic urticaria specific IgE antibodies to autologous sweat antigens or skin resident fungi have been reported.^23^, ^24^

The possible involvement of antibodies in the pathogenesis of CSU and CIndU has fueled the search of therapeutic alternatives for cases resistant to conventional approaches. Blocking BTK signaling leads to impairment of B cell activation and diminishing antibody production and to a decrease in FcεR1‐mediated basophil and mast cell activation and degranulation. Remibrutinib, an oral covalent BTK inhibitor, has shown in a phase I trial to be very effective in preventing basophil activation in ex vivo assays using the upregulation of CD63 and CD203c markers as a functional read‐out.^7^ Furthermore, a recent phase IIb trial has confirmed the effectiveness of remibrutinib in rapidly controlling the signs (hives) and symptoms (itching) in patients with moderate to severe CSU.^7^

In this work we provide biological evidence of the ability of remibrutinib at inhibiting the degranulation of human basophils obtained from healthy donors or CD34^+^ derived mast cells triggered by the serum of patients with CU. Remibrutinib blocks the upregulation of CD63 both in CD34^+^ mast‐cells and blood basophils in response CSU and CIndU sera independently of the baseline FcεR1 expression and omalizumab response. Interestingly, remibrutinib blocks the upregulation of CD63 in response to factors present in the sera of either CSU and CIndU patients suggesting that both antibody‐dependent and antibody‐independent mechanisms^20^, ^21^, ^25^ may be play a role in the hives etiopathogenesis. The mechanism through which remibrutinib would act more globally by inhibiting mast cell and basophil activation requires further investigation. Similarly, the reactivity of mast cells to the serum stimuli in general seems to be higher than in basophils, despite their lower FcεR1 expression and the non‐specific occupancy of the receptor. In contrast, using antibodies directed specifically to the receptor to induce cell activation, the response is the opposite, with basophils overwhelmingly more responsive than mast cells, probably due to their higher FcεR1 expression. It is possible that different types of triggers co‐exist in CU patients for example, coagulation factors acting through C5a,^26^ involving mast cells, basophils, and other different cell types as lymphocytes or eosinophils active in the hive pathogenesis. Depending on the relative contribution of each of these factors, one or the other treatment may be more effective. If autoallergy is dominant or in patients with high FcεR1expression, omalizumab may be the best choice, but if other mechanisms are at work, other drugs such as remibrutinib, targeting intracellular signaling cascades, may be necessary to modulate the response of effector cells.

Anti‐IgE antibodies are an effective and safe biological therapy option for antihistamine‐resistant CU patients once FcεRI crosslink with IgE‐autoallergen, IgE–IgG, IgG, or IgM.^27^ However, there are still patients who do not benefit from this approach.^3^ We have found that serum from omalizumab responders and from non‐responders are similar in their ability to activate basophils and mast cells suggesting that similar triggers may be acting in these two phenotypes. However, patient's serum may contain a spectrum of factors that can activate effector cells. To know the relative contribution of these factors may be useful to select the best treatment. Indeed, our results show that remibrutinib works on basophils and very importantly also on mast cells and that BTK inhibition may reduce the activation of effector cells induced by IgE‐dependent and independent mechanisms.

In this study it was shown first that remibrutinib inhibits experimentally mast cell and basophil activation induced by IgE cross‐linking. Compared with sera obtained from HCs the serum from CU patients has a greater ability to induce mast cell and basophil degranulation. The activation of blood basophils and mast cells in culture by patient CU sera (CSU and CindU) is not linked to the baseline FcεR1expression and omalizumab responsiveness. Remibrutinib inhibits in vitro basophil and mast cell activation induced by CU serum (CSU and CIndU) independently of FcεR1expression and omalizumab response.

To sum up, in the current study we describe that, in vitro, mast cell activation induced by serum from CSU and CIndU patients can be inhibited by remibrutinib. Very importantly, serum activity from both, omalizumab responders and non‐responders could be equally inhibited by remibrutinib.

## AUTHOR CONTRIBUTIONS

**Ramon Gimeno**: Conceptualization (equal); data curation (equal); formal analysis (equal); investigation (equal); methodology (equal). **Clara Ribas‐Llauradó**: Data curation (equal); formal analysis (equal); investigation (equal); methodology (equal). **David Pesque**: Formal analysis (equal); investigation (equal). **Evelyn Andrades**: Data curation (equal); formal analysis (equal); investigation (equal); methodology (equal); project administration (equal). **Bruno Cenni:** Funding acquisition (equal); resources (equal); supervision (equal); validation (equal). **Barbara Ambros**: Resources (equal); supervision (equal). **Ramon Pujol**: Supervision (equal); validation (equal). **Ana M. Gimenez‐Arnau**: Conceptualization (equal); data curation (equal); formal analysis (equal); funding acquisition (equal); investigation (equal); methodology (equal); project administration (equal); resources (equal); supervision (equal); validation (equal).

## CONFLICTS OF INTEREST STATEMENT

Ana M. Giménez‐Arnau: Medical Advisor for Uriach Pharma/Neucor, Genentech, Novartis, FAES, GSK, Sanofi–Regeneron, Amgen, Thermo Fisher Scientific, Almirall, Celldex, Leo Pharma Research Grants supported by Uriach Pharma, Novartis, Grants from Instituto Carlos III‐FEDER Educational activities for Uriach Pharma, Novartis, Genentech, Menarini, LEO‐PHARMA, GSK, MSD, Almirall, Sanofi, Avene. Bruno Cenni and Barbara Ambros are Novartis employees. The rest of authors declare that there is no conflict of interest concerning the present work.

## Supporting information

Supporting Information S1
