# Supplementary material for: Remibrutinib inhibits hives effector cells stimulated by serum from chronic urticaria patients independently of FcεR1 expression level and omalizumab clinical response
Source: Clin Transl Allergy. 2023 Mar 4;13(3):e12227. doi: 10.1002/clt2.12227 (PMC9985467; doi:10.1002/clt2.12227)

**SUPPLEMENTARY FIGURES**

**Supplementary Figure 1**. **Generation and differentiation of human mast cells derived from CD34^+^ cord blood hematopoietic progenitors**. **A**. Graphical representation of the culture protocol. **B**. Representation of the flow cytometry analysis of the cells harvested from the cultures at the indicated times showing the acquisition of the c-kit^+^/FcεR1a^+^ phenotype characteristic of mast cells. In the last plot it is shown the effect of the sensitization step on the expression levels of FcεR1a.


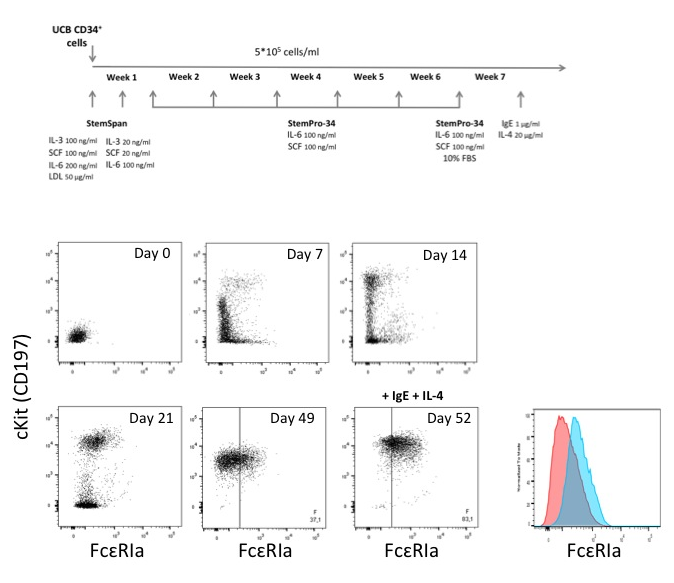


A

B

**Supplementary Figure 2. Mast cells and basophils show different sensitivity to activation by serum factors and inhibition by remibrutinib.** CD34^+^-derived mast cells or blood basophils were incubated with serum obtained from chronic urticaria patients and the upregulation of CD63 established in the relevant population by using specific monoclonal antibodies and flow cytometry. Percentages of CD63-expressing cells are referred to the maximum activation obtained with a positive control. On the left panel the effect of remibrutinib on mast cells and basophils is shown. Mann-Whitney U test: *P<0.05, ***P<0.01.


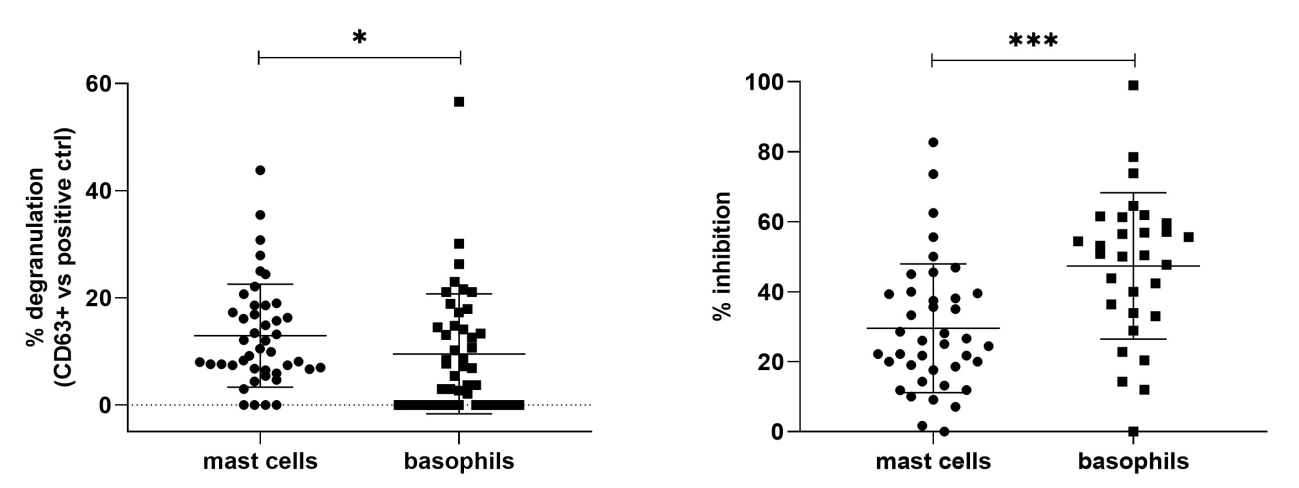

Supplement: Supplementary file 1 — Supporting Information S1 [file CLT2-13-e12227-s001.docx]
